# Supplementary material for: Integrated Bioinformatics-Based Subtractive Genomics Approach to Decipher the Therapeutic Drug Target and Its Possible Intervention against Brucellosis
Source: Bioengineering (Basel). 2022 Nov 1;9(11):633. doi: 10.3390/bioengineering9110633 (PMC9687753; doi:10.3390/bioengineering9110633)
Supplement: Supplementary file 1 [file bioengineering-09-00633-s001.zip › bioengineering-1946749-supplementary.pdf]

Integrated Bioinformatics Based Subtractive Genomics Approach to Decipher the Therapeutic Drug Target and its Possible Intervention Against Brucellosis

Kanwal Khan [1], Munirah Sulaiman Othman Alhar[2], Muhammad Naseer Abbas [3], Syed Qamar Abbas[4], Mohsin Kazi[5], Saeed Ahmad Khan [6,7], Abdul Sadiq[8], Syed Shams ul Hassan[9,10]*, Simona Bungau[11]*, Khurshid Jalal[12]*

1 Dr. Panjwani Center for Molecular Medicine and Drug Research, International Center for Chemical and Biological Sciences, University of Karachi, Pakistan
2 Department of Chemistry, College of Science, University of Ha'il, Ha'il 81451, Saudi Arabia
3 Department of Pharmacy, Kohat University of Science and Technology, Kohat KP Pakistan 26000
4 Department of Pharmacy, Sarhad University of science and technology, Peshawar
5 Department of Pharmaceutics, College of Pharmacy, King Saud University, P.O. Box 2457, Riyadh 11451, Saudi Arabia
6 Department of Pharmacy, Kohat University of Science and Technology, 26000 Kohat, Pakistan
7 Division of Molecular Pharmaceutics and Drug Delivery, The University of Texas at Austin, 2409 University Ave., Austin, Texas, USA
8 Department of Pharmacy, Faculty of Biological Sciences, University of Malakand, Chakdara, 18000 Dir (L), KP, Pakistan
9 Shanghai Key Laboratory for Molecular Engineering of Chiral Drugs, School of Pharmacy, Shanghai Jiao Tong University, Shanghai 200240, China
10 Department of Natural Product Chemistry, School of Pharmacy, Shanghai Jiao Tong University, Shanghai 200240, China
11 Department of Pharmacy, Faculty of Medicine and Pharmacy, University of Oradea, 410028 Oradea, Romania
12 HEJ Research Institute of Chemistry International Center for Chemical and Biological Sciences, University of Karachi, Pakistan
* Correspondence: khurshid@iccs.edu (K.J); Shams1327@yahoo.com (S.S.U.H), Simonabungau@gmail.com (S.B)

Supplementary Table S1: Common Metabolic Pathways of Brucella suis and Human host			
S. No	Common Metabolic Pathways	Pathways ID	Number of Proteins
1	Glycolysis	Bms00010	33
2	Citric acid cycle	Bms00020	24
3	Pentose phosphate pathway	Bms00030	18
4	Pentose and glucoronate interconversion	Bms00040	12
5	Fructose and mannose metabolism	Bms00051	17
6	Galactose metabolism	Bms00052	8
7	Ascorbate and adorate metabolism	Bms00053	5
8	Starch and sucrose metabolism	Bms00500	6
9	Amino sugar and nucleotide metabolism	Bms00520	31
10	Pyruvate metabolism	Bms00620	47
11	Glyoxylate and dicarboxylate metabolism	Bms00630	36
12	Propanoate metabolism	Bms00640	30
13	Butanoate metabolism	Bms00650	24
14	Inositol phosphate metabolism	Bms00562	8
15	Oxidative phosphorylation	Bms00190	52
16	Nitrogen metabolism	Bms00910	18
17	Sulphur metabolism	Bms00920	18
18	Fatty acid biosynthesis	Bms00061	23
19	Fatty acid degradation	Bms00071	21
20	Fatty acid elongation	Bms00070	1
21	Synthesis and degradation ketone bodies	Bms00072	4

22	Glycerolipid metabolism	Bms00561	9
23	Glycerophospholipid metabolism	Bms00564	13
24	Alpha-linolenic acid metabolism	Bms00592	1
25	Biosynthesis of unsaturated fatty acid	Bms01040	3
26	Purine metabolism	Bms00230	53
27	Pyrimidine metabolism	Bms00240	27
28	Alanine aspartate and glutamate metabolism	Bms00250	27
29	Glycine serine and threonine metabolism	Bms00260	32
30	Cysteine and methionine metabolism	Bms00270	23
31	Valine leucine and isoleucine degradation	Bms00280	31
32	Valine leucine and isoleucine biosynthesis	Bms00290	13
33	Lysine degradation	Bms00310	19
34	Arginine biosynthesis	Bms00220	22
35	Arginine and proline metabolism	Bms00330	25
36	Histidine metabolism	Bms00340	19
37	Tyrosine metabolism	Bms00350	16
38	Phenyl alanine metabolism	Bms00360	14
39	Tryptophane metabolism	Bms00380	17
40	Phenylalanine tyrosine and tryptophan biosynthesis	Bms00400	20
41	Beta alanine metabolism	Bms000410	13
42	Taurine and hypo taurine metabolism	Bms00430	2
43	Phosphonate and phophenate metabolism	Bms00440	2
44	Selenocompound metabolism	Bms00450	8

45	D-glutamine and D-glutamate metabolism	Bms00471	5
46	D-arginine and D-ornithine metabolism	Bms00472	1
47	Glutathione metabolism	Bms00480	18
48	Thymine metabolism	Bms00730	12
49	Riboflavin metabolism	Bms00740	7
50	Vitamins B6 metabolism	Bms00750	6
51	Nicotinate and nicotinamide metabolism	Bms00760	15
52	Pantothenate and CoA biosynthesis	Bms00770	20
53	Biotin metabolism	Bms00780	18
54	Lipoic acid metabolism	Bms00785	2
55	Folate biosynthesis	Bms00790	21
56	One-carbon pool by folate	Bms00670	11
57	Porphyrin and chlorophyll metabolism	Bms00860	41
58	Ubiquinone and another terpenoid-quinone biosynthesis	Bms00130	9
59	Terpenoid backbone biosynthesis	Bms00900	11
60	RNA polymerase	Bms03020	4
61	Amino-acyl tRNA biosynthesis	Bms00970	26
62	Protein export	Bms03060	16
63	Sulphur relay system	Bms04122	10
64	RNA degradation	Bms03018	14
65	DNA replication	Bms03030	9
66	Base excision repair	Bms03410	12
67	Nucleotide excision repair	Bms03420	7

68	Mismatch repair	Bms03430	17
69	Homologous recombination	Bms03440	21
70	ABC transporter	Bms02010	188

Supplementary Table S2: 42 shortlisted drug like targets against B. suis			
**1**	WP_002963761.1	response regulator transcription factor	Cytoplasmic 9.97
**2**	WP_002964109.1	D-alanyl-D-alanine carboxypeptidase	Cytoplasmic Membrane 9.97
**3**	WP_002964699.1	response regulator transcription factor	Cytoplasmic 9.97
**4**	WP_002965100.1	D-alanyl-D-alanine carboxypeptidase	Cytoplasmic Membrane 9.97
**5**	WP_002965156.1	response regulator transcription factor	Cytoplasmic 9.97
**6**	WP_002965207.1	phosphate regulon transcriptional regulator PhoB	Cytoplasmic 9.97
**7**	WP_002965384.1	global response regulator transcription factor RegA	Cytoplasmic 9.97
**8**	WP_002966022.1	response regulator	Cytoplasmic 9.97
**9**	WP_002966153.1	response regulator transcription factor	Cytoplasmic 9.97
**10**	WP_002966190.1	response regulator transcription factor	Cytoplasmic 9.97
**11**	WP_002966355.1	sensor histidine kinase N-terminal domain-containing protein	Cytoplasmic Membrane 7.88
**12**	WP_002966460.1	HTH-type quorum sensing-dependent transcriptional regulator VjbR	Unknown
**13**	WP_002966538.1	two-component sensor histidine kinase	Cytoplasmic Membrane

			10.00
**14**	WP_002966632.1	D-xylose ABC transporter substrate-binding protein	Periplasmic 9.76
**15**	WP_002968915.1	ABC transporter substrate-binding protein	Periplasmic 9.76
**16**	WP_002970053.1	PAS domain-containing sensor histidine kinase	Cytoplasmic Membrane 10.00
**17**	WP_002971389.1	response regulator transcription factor	Cytoplasmic 9.97
**18**	WP_002971443.1	PAS domain-containing hybrid sensor histidine kinase/response regulator	Cytoplasmic Membrane 9.99
**19**	WP_002971701.1	LuxR family transcriptional regulator	Cytoplasmic 9.26
**20**	WP_004681910.1	PleD family two-component system response regulator	Cytoplasmic 9.97
**21**	WP_004681922.1	IclR family transcriptional regulator	Cytoplasmic 9.97
**22**	WP_004684524.1	LacI family DNA-binding transcriptional regulator	Cytoplasmic 9.97
**23**	WP_004684628.1	LysR family transcriptional regulator	Cytoplasmic 9.97
**24**	WP_004684782.1	ABC transporter substrate-binding protein	Periplasmic 9.76
**25**	WP_004686048.1	sn-glycerol-3-phosphate ABC transporter substrate-binding protein UgpB	Periplasmic 10.00
**26**	WP_004687154.1	nickel ABC transporter, nickel/metallophore periplasmic binding protein	Periplasmic 10.00

**27**	WP_004689042.1	amino acid ABC transporter substrate-binding protein	Periplasmic 9.44
**28**	WP_004690331.1	D-ribose ABC transporter substrate-binding protein	Periplasmic 9.76
**29**	WP_004690544.1	two-component sensor histidine kinase	Cytoplasmic Membrane 7.88
**30**	WP_004690676.1	response regulator transcription factor	Cytoplasmic 9.97
**31**	WP_004690853.1	D-alanyl-D-alanine carboxypeptidase	Cytoplasmic Membrane 9.97
**32**	WP_004690877.1	sigma-54-dependent Fis family transcriptional regulator	Cytoplasmic 9.97
**33**	WP_004691511.1	isocitrate lyase	Cytoplasmic 9.97
**34**	WP_006133828.1	ABC transporter substrate-binding protein	Periplasmic 10.00
**35**	WP_006189967.1	LacI family DNA-binding transcriptional regulator	Cytoplasmic 9.97
**36**	WP_006190489.1	D-alanyl-D-alanine carboxypeptidase	Cytoplasmic Membrane 9.82
**37**	WP_006191518.1	IclR family transcriptional regulator	Cytoplasmic 9.97
**38**	WP_006192075.1	Fe(3+) ABC transporter substrate-binding protein	Periplasmic 9.76
**39**	WP_006192133.1	ABC transporter substrate-binding protein	Periplasmic 10.00
**40**	WP_006278874.1	peptide ABC transporter substrate-binding protein	Periplasmic 10.00

**41**	WP_011068960.1	multidrug efflux RND transporter outer membrane subunit BepC	Outer Membrane 10.00
**42**	WP_002966536.1	response regulator	Cytoplasmic 9.97
